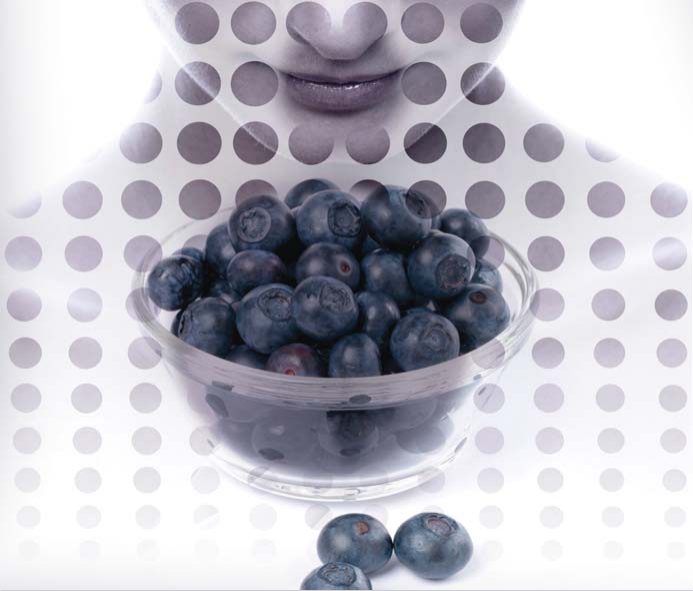# Nutrigenomics: The Genome–Food Interface

**DOI:** 10.1289/ehp.115-a582

**Published:** 2007-12

**Authors:** M. Nathaniel Mead

Efforts to unveil the etiology of human disease often recapitulate the nature versus nurture debate. But today’s biologists concede that neither nature nor nurture alone can explain the molecular processes that ultimately govern human health. The presence of a particular gene or mutation in most cases merely connotes a predisposition to a particular disease process. Whether that genetic potential will eventually manifest as a disease depends on a complex interplay between the human genome and environmental and behavioral factors. This understanding has helped spawn numerous multidisciplinary gene-based approaches to the study of health and disease.

One such endeavor is nutrigenomics, the integration of genomic science with nutrition and, when possible, other lifestyle variables such as cigarette smoking and alcohol consumption. Although genes are critical for determining function, nutrition modifies the extent to which different genes are expressed and thereby modulates whether individuals attain the potential established by their genetic background.

Nutrigenomics therefore initially referred to the study of the effects of nutrients on the expression of an individual’s genetic makeup. More recently, this definition has been broadened to encompass nutritional factors that protect the genome from damage. Ultimately, nutrigenomics is concerned with the impact of dietary components on the genome, the proteome (the sum total of all proteins), and the metabolome (the sum of all metabolites). As in pharmacogenomics, where a drug will have diverse impacts on different segments of the population, researchers recognize that only a portion of the population will respond positively to specific nutritional interventions, while others will be unresponsive, and still other could even be adversely affected.

## A Focus on Polymorphisms

Numerous studies in humans, animals, and cell cultures have demonstrated that macronutrients (e.g., fatty acids and proteins), micronutrients (e.g., vitamins), and naturally occurring bioreactive chemicals (e.g., phytochemicals such as flavonoids, carotenoids, coumarins, and phytosterols; and zoochemicals such as eicosapentaenoic acid and docosahexaenoic acid) regulate gene expression in diverse ways. Many of the micronutrients and bioreactive chemicals in foods are directly involved in metabolic reactions that determine everything from hormonal balances and immune competence to detoxification processes and the utilization of macronutrients for fuel and growth. Some of the biochemicals in foods (e.g., genistein and resveratrol) are ligands for transcription factors and thus directly alter gene expression. Others (e.g., choline) alter signal transduction pathways and chromatin structure, thus indirectly affecting gene expression.

There is increasing evidence that genome instability, in the absence of overt exposure to genotoxicants,is itself a sensitive marker of nutritional deficiency.

–Michael Fenech, CSIRO Genome Health and Nutrigenomics Laboratory

Much of the nutrigenomic focus has been on single-nucleotide polymorphisms (SNPs), DNA sequence variations that account for 90% of all human genetic variation. SNPs that alter the function of “housekeeping genes” involved in the basic maintenance of the cell are assumed to alter the risk of developing a disease. Dietary factors may differentially alter the effect of one or more SNPs to increase or decrease disease risk.

An elegant example of a diet–SNP interaction involves the common C677T polymorphism of the methylenetetrahydrofolate reductase (*MTHFR*) gene. This variant causes MTHFR enzyme activity to slow down. This results in reduced capacity to use folate (or folic acid) to convert homocysteine to methionine and thence to the *S*-adenosyl-methionine required for the maintenance methylation of cytosine in DNA and control of gene expression, among many other reactions. But the same variant also may increase the form of folate that can be used to make thymidine, one of the bases in DNA, and to prevent mutagenic uracil from being incorporated instead. This shift in methylation status may explain why in a low-folate environment (for example, where there is low intake of folate-rich vegetables such as spinach and asparagus or a lack of supplemental folate) homozygous carriers of the C677T polymorphism may be more prone to developmental defects but at the same time could be protected against certain cancers.

The key point here is that the activity of the reaction catalyzed by the *MTHFR* gene can be modified depending on the amount of two essential nutrients: folate, which is the substrate for *MTHFR*, and riboflavin, a cofactor of *MTHFR*. “Therefore, the risks associated with *MTHFR* activity can be markedly modified, for better or for worse, depending on fortification and supplementation strategies,” says Michael Fenech, a research scientist at the CSIRO Genome Health and Nutrigenomics Laboratory in Adelaide, Australia. “For example, in those countries where mothers are required to supplement with high-dose folic acid to prevent neural tube defects in the infant, this practice may actually allow more babies to be born with the *MTHFR* C677T [polymorphism].” These children would be less able to convert folate to a usable form. On the other hand, if the dietary environment in which these individuals have to grow is low in folate and riboflavin, then they may struggle to survive in good health.

The field of nutrigenomics could not have been launched without the recent development of high-throughput -omic (genomic, transcriptomic, proteomic, and metabolomic) technologies. “These technologies enable us to identify and measure many molecules of each type at one time,” says Jim Kaput, director of the newly established Division of Personalized Nutrition and Medicine at the FDA National Center for Toxicological Research. “In the realm of genomics, for example, we can now measure many variations in DNA, including tens of thousands of single-nucleotide polymorphisms and copy number variants, as well as many RNA molecules. This is crucial, since most cases of chronic diseases are not caused by mutations in single genes but rather by complex interactions among variants of several . . . genes.”

These technologies currently enable identification of up to 500,000 SNPs per individual. Whereas nucleic acids can be analyzed with either sequencing or hybridization technologies, protein and metabolites may require slightly different techniques and equipment depending upon the type of protein and chemical nature of the metabolite. Nevertheless, Kaput says, the end result using various -omic technologies is an incredibly detailed window into the molecular makeup of each individual.

Meanwhile, nutritional biochemists have been busily cataloguing factors in food, including dozens of essential nutrients and tens of thousands of bioactive substances, that can be correlated with molecular patterns identified through the various -omic technologies. The intersection of the genomic and nutritional domains will require sophisticated analytic techniques and, in Kaput’s opinion, the open sharing of scientific research findings worldwide because of the value derived from studying genomic and nutritional patterns in different populations and ethnic groups.

## The Sweet Spot for Genomic Health

Not only the expression of genes but also the physical integrity and stability of the genome—what has been referred to as “genome health”—is to a large degree determined by a steady supply of specific nutrients. “There is increasing evidence that genome instability, in the absence of overt exposure to genotoxicants, is itself a sensitive marker of nutritional deficiency,” says Fenech.

Fenech originated the concept of “genome health nutrigenomics,” the science of how nutritional deficiency or excess can cause genome mutations at the base sequence or chromosomal level. “The main goal of this particular research discipline is to define the optimal dietary intake and tissue culture medium concentration to maintain damage to the genome at its lowest possible level *in vivo* and *in vitro*, respectively,” says Fenech. “This is critically important because increased damage to the genome is among the fundamental causes of infertility, developmental defects, cancer, and neurodegenerative diseases.” By the same token, the selective use of genome-protective nutrients in individuals with specific gene variants could potentially result in improved resistance toward these major diseases. Fenech believes we need to start viewing foods and diets in terms of their content of genome-protective nutrients.

Folate is among the nutrients most often cited as critical to genomic stability. Controlled intervention study data published in the July 1998 issue of *Carcinogenesis* and the April 2001 issue of *Mutation Research* indicate that a folate intake greater than 200 μg/day is required for chromosomal stability. Fenech’s team has shown that reducing plasma folate concentration from 120 to 12 nmol/L *in vitro*, which is considered to be within the equivalent adequate range *in vivo*, causes as much genome damage as that induced by an acute exposure to 0.2 Gy of ionizing radiation. “We concluded that even moderate folate deficiency within the physiological range causes as much DNA damage in cultured lymphocytes as ten times the annual allowed limit of exposure to X rays and other forms of low linear energy transfer ionizing radiation for the general population,” says Fenech. He points out that the typical plasma folate concentration for most populations is only 10–30 nmol/L, a level adequate to prevent anemia “but apparently insufficient to minimize chromosomal damage.”

In the May 2005 issue of *Carcinogenesis* Fenech and his colleagues identified nine key nutrients that may affect genomic integrity in various ways. When consumed in increasing amounts in food, six of these nutrients (folate, vitamin B_12_, niacin, vitamin E, retinol, and calcium) are associated with a reduction in DNA damage, whereas three others (riboflavin, pantothenic acid, and biotin) are associated with an increase in DNA damage to the same extent observed with occupational exposure to genotoxic and carcinogenic chemicals. “These observations indicate that nutritional deficiency or excess can cause DNA damage on its own and that the effects are of the same magnitude as that of many common environmental toxicants,” Fenech says.

Paul Soloway, a nutrition professor at Cornell University, points out that characterizing diets or specific nutrients as being genome-damaging or genome-protecting on the basis of *in vitro* studies overlooks the variations in benefits that exist over a lifetime, particular relative to the timing of disease onset. Moreover, nutritionists have long understood that the optimal requirements for many nutrients fall within a range between deficiency and toxicity. In an environment of vitamin fortification and supplementation, Fenech’s findings may compel health officials to be more vigilant about not exceeding levels that could be harmful to the genome or that might even promote the growth of latent cancers. As an example of how controversial these concerns may be, some studies have reported protective benefits from folate for initiation of colorectal cancer, whereas others have found that this nutrient may promote the growth of this cancer once it is established.

Defining the optimal concentration of micronutrients required to maintain cells in a genomically stable state remains one of the main challenges for nutrigenomics researchers. This challenge becomes magnified in the context of requirements for diverse genetic backgrounds. Fenech cites the example of individuals who show inherited defects in DNA repair: these individuals may be more vulnerable to the DNA-damaging effects of moderate folate deficiency than those who do not have such defects.

There are thousands of DNA alterations in each human cell daily; if not efficiently repaired, our genome would rapidly be destroyed. Diet and lifestyle are major mediating factors in this equation. For example, DNA damage is accelerated by oxidative stressors such as tobacco smoke, strenuous exercise, and a high-fat diet, according to a study in the September 2002 issue of *Carcinogenesis*. On the flip side, diets low in fat and/or high in cruciferous vegetables have been shown to lower the oxidative DNA damage rate in humans, as indicated by reduced urinary excretion of 8-oxo-7,8-dihydro-2'-deoxyguanosine (8-oxodG). In other reports, the dietary intake of vitamin C determined the concentration of 8-oxodG in human sperm DNA, while dietary fish oil and calcium reduced oxidative DNA damage rate in colonic epithelial cells.

When it comes to maintaining genomic integrity, epigenetic changes such as those involving DNA and histone modifications are as profound as the genetic ones. “The loss of normal epigenetic states can lead to genomic rearrangements and increased failure of mismatch repair,” says Soloway. The example of folate and *MTHFR* helps highlight the dynamic interplay between the genome and epigenome, he says: “Because there are considerable epigenetic influences of nutrients such as folate, one of the ways by which alleles of *MTHFR* might control nutrient-related phenotypes is through epigenetic mechanisms.” Changes in the epigenome in response to dietary factors may often precede changes in the genome, and yet those genomic changes help solidify the emergence of new epigenetic patterns within the organism.

In addition to folate, various antioxidant nutrients and phytochemicals are known to enhance DNA repair and reduce oxidative DNA damage, and such dietary contributions could theoretically compensate for inherited defects in repair mechanisms. Also, individuals with inherited polymorphisms that lower the activity of antioxidant enzyme systems such as manganese superoxide dismutase and glutathione peroxidase may have a higher requirement for dietary antioxidants to prevent DNA damage or cancer risk.

Although it is tempting to focus on single-nutrient effects such as the folate example mentioned above, nutrigenomics researchers contend that the real focus should be on the impact of multiple nutritional imbalances (both excess and deficiency) on the genome. In their May 2005 *Carcinogenesis* article, which described a study of 190 healthy men and women with an average age of 48 years, Fenech and his colleagues showed that high intakes of various B vitamins—riboflavin, pantothenic acid, and biotin—actually increased micronucleus frequency in lymphocytes, a standard measure of genome damage.

Going further, they studied the combined effects of calcium or riboflavin with different levels of folate intake, since earlier studies had indicated that these dietary factors tend to interact in modifying the risk of cancer, osteoporosis, and hip fractures. Increasing one’s calcium intake further enhanced the genome-protective effect of a high-folate diet whereas a high riboflavin intake further exacerbated genome damage associated with a low-folate diet. This is consistent with epidemiologic studies showing that cancer rates tend to be higher among populations that consume more red meat (which is very high in riboflavin), more alcohol (which depletes folate), and fewer vegetables (a rich source of folate).

The promise of nutrition-modulated DNA repair strategies has attracted the attention of cancer researchers in particular. “Dietary factors can act to stabilize the genome once genetic abnormalities have occurred,” says gastroenterologist Graeme Young, who directs the Flinders Centre for Innovation in Cancer in Adelaide, Australia. “The traditional diet–genome approach has related protection to dietary lifestyle and germline genotype,” he says. “Here we are discussing dietary interaction with the abnormal genome in potentially precancerous cells.” Young and his colleagues are now planning to explore the capacity of dietary factors to regulate DNA repair mechanisms.

## Nutrigenomic Links to Chronic Disease

Ben van Ommen, director of the European Nutrigenomics Organization, and colleagues hypothesize that all diseases can be reduced to imbalances in four overarching processes: inflammatory, metabolic, oxidative, and psychological stress. Diseases arise because of genetic predispositions to one or more of these stressors. Nutrigenomics represents a major effort to improve our understanding of the role of nutrition and genomic interactions in at least the first three of these areas, says Kaput. In time, he adds, we will see important contributions from nutrigenomics for the prevention of many common modern maladies, including obesity, diabetes, cardiovascular disease, cancer, inflammatory disorders, age-related cognitive disorders, visual function, and of course many vitamin deficiency problems.

Diabetes, obesity, and cardiovascular diseases have been referred to by medical anthropologists and others as “diseases of civilization.” The reason is simple: when aboriginal populations begin to adopt a high-sugar, high-fat “Western diet” for the first time, obesity and diabetes suddenly begin to appear in those populations and typically increase at rates commensurate with the adoption of the new diet. Such observations have been dramatically borne out in studies of the Pima Indians of Arizona and the indigenous people of Hawaii. In both instances, the abandonment of the traditional plant-rich, high-fiber diet was followed by skyrocketing rates of diabetes, obesity, and later cancer.

Dietary factors can act to stabilize the genome once genetic abnormalities have occurred. The traditional diet–genome approach has related protection to dietary lifestyle and germline genotype. Here we are discussing dietary interaction with the abnormal genome in potentially precancerous cells.

–Graeme Young, Flinders Centre for Innovation in Cancer

Over the course of human evolution, diet has profoundly molded human metabolic capacities and thus paved the way for the emergence of modern diseases. From an evolutionary perspective, diet is a limiting factor that imposes selective pressures on a population, much like other environmental factors. Some genotypes within a population are associated with higher nutrient needs, and when those needs are not met, there will be selection against those particular genotypes. However, when those needs are met—for example, the need for extra calories from carbohydrates and dietary fat—the gene that confers the high nutrient requirement will then persist in the population. This could well be the case for genes linked with obesity and diabetes.

Soloway notes that in cases where certain gene alleles confer some selective advantage, high levels of the required nutrient can actually lead to an expanded frequency of those alleles in a population. “In such cases, nutrient availability can provide a selective pressure that drives genotypic shifts in a population,” he says.

From the nutrigenomic perspective, diabetes and obesity are both the result of an imbalanced diet interacting with genes that were once functional and adaptive in an earlier phase of human evolution, when food was less abundant. In the modern context, these same genes are considered to code for hormonal or metabolic tendencies that have become maladaptive and pathological in the modern environment. Risk of developing these diseases is thought to be modulated by genetic susceptibility differences among ancestral groups to the effect of the Western diet in precipitating insulin resistance.

In addition, says Lynn Ferguson, a nutrition professor at the University of Auckland in New Zealand and program leader of the New Zealand National Centre for Research Excellence in Nutrigenomics, “the control of food intake is profoundly influenced by gene variants encoding taste receptors or those encoding a number of peripheral signaling peptides such as insulin, leptin, ghrelin, cholecystokinin, and corresponding receptors. Total dietary intake, and the satiety value of various foods, will profoundly modify the impact of these genes.” In volume 10, number 2 (2006) of *Molecular Diagnosis & Therapy*, Ferguson cites studies that have linked five common SNPs with increased obesity risk and resistance to weight reduction. “These SNPs represent promising targets for future nutrigenomic studies of people at risk for obesity,” she says. Taken together, these findings provide a strong scientific rationale for avoiding a generic, one-size-fits-all approach to the problem of obesity.

Given that obesity is itself a risk factor for diabetes, cardiovascular disease, and various cancers, it is worthwhile to focus on the nutrigenomic aspects of this disease. A study conducted at the University of Navarra in Pamplona, Spain, and published in the August 2003 issue of the *Journal of Nutrition* showed that women with a *Glu27* variant and a carbohydrate intake constituting more than 49% of total caloric consumption had a nearly three-fold increase in their risk of developing obesity. Importantly, an alternative variant of that same gene was not linked with a greater obesity risk in relation to the same carbohydrate–calorie intake levels. This could help explain why some women on high-carbohydrate diets gain weight while others do not.

Abdominal obesity, independent of generalized adiposity, predicts insulin resistance, type 2 diabetes, dyslipidemia, and cardiovascular disease. Endocrinologist Jerry Greenfield and colleagues at St. Vincent’s Hospital in Sydney, Australia, recently reported that high polyunsaturated fat intake was associated with lower levels of abdominal fat in women at low genetic risk for abdominal obesity but not in women at high genetic risk. Also, a moderately high alcohol intake (1–1.5 drinks per day) was associated with approximately 20% less abdominal fat than lower intakes, but only in women genetically predisposed to abdominal obesity. This study, published in the November 2003 *Journal of Clinical Endocrinology and Metabolism*, indicates that various gene–diet interactions could be a key part of the abdominal obesity equation.

Diet–gene interactions are highly complex and hard to predict, thus demonstrating the need for highly controlled genotypes and environmental conditions that allow for identifying different regulatory patterns based on diet and genotype. The challenges we now face may ultimately require a nutrigenomics project on the scale of the Human Genome Project in order to identify genes that cause or promote chronic disease and the nutrients that regulate or influence the activity of these genes.

–Jim Kaput, FDA National Center for Toxicological Research

The *APOE* gene offers another example of how certain polymorphisms may predispose their bearers to chronic diseases. Each of three phenotypes carries a different probability of cardiovascular disease risk and responds differently to lifestyle and environmental factors, including dietary variables such as the amount and type of dietary fat. Most people in the United States have the *APOE3* phenotype and respond favorably to a lower intake of dietary fat and regular exercise: their cholesterol levels drop and overall cardiovascular health improves. However, about 20% of the U.S. population carries at least one variant denoted as *APOE-*ε4, a polymorphism associated with elevated total cholesterol level, as well as an increased risk of both type 2 diabetes and Alzheimer disease. The SNP also abrogates the protective effects seen with moderate alcohol consumption and greatly increases the cardiovascular risks associated with smoking, dramatically boosting the risk of heart attack in such individuals.

“The implication here is that anyone with this genotype should be rigorously attentive to their diet and lifestyle,” says Ferguson. “These individuals should avoid smoking and alcohol while undertaking exercise and eating a diet low in saturated fat. Nonetheless, at present, very few people are aware of their *APOE* genotype.” Lack of the awareness of such SNP–diet–lifestyle interactions is not only a drawback for public health education, but also may result in null findings in epidemiologic studies when in fact certain segments of the study population are highly vulnerable to diseases that are linked with a given SNP.

## Future Research Directives and Challenges

Identifying the SNP–diet and SNP–nutrient interactions that cause chronic disease is challenging because of the complexities inherent in studying genotypes and in assessing dietary and nutrient intakes. At this time, few if any of the SNP–diet associations that have been reported in epidemiologic studies have been replicated, and many have been plagued by a lack of appropriate statistical power and other methodologic problems. Ultimately, because many cases of chronic diseases are influenced by different diets, nutrition–genome interactions will not be found unless diet and genotype are controlled and changed in the experimental design (same diet with different genotypes, and different genotypes with the same diet).

“Diet–gene interactions are highly complex and hard to predict, thus demonstrating the need for highly controlled genotypes and environmental conditions that allow for identifying different regulatory patterns based on diet and genotype,” Kaput says. “The challenges we now face may ultimately require a nutrigenomics project on the scale of the Human Genome Project in order to identify genes that cause or promote chronic disease and the nutrients that regulate or influence the activity of these genes.”

Because human intervention studies are costly and difficult to conduct, observational studies (which detect associations, not causal relationships) will likely continue to dominate the epidemiologic approach to nutrigenomics. For interventional and mechanistic data, *in vivo* animal studies will be heavily favored because lab animals can be selected for minimal genetic variation and shorter life spans. Moreover, it is much easier to control and monitor the dietary intakes of animals than those of humans.

Kaput notes that assessments of dietary intake, albeit mundane to the outside world, may represent one of the biggest impediments to the success of large-scale human nutrigenomic studies. “Quantifying food intake is challenging because free-living humans simply do not regard daily life as a science experiment where the amount and type of food is accurately recorded,” he says. To avoid measurement problems such as misclassification, more reliable measurement tools for assessing nutrient intake will be needed in the years ahead.

Proponents of nutrigenomics research have cited the population-wide prevention and treatment of vitamin deficiency as a top public health priority. Since vitamin deficiencies are highly prevalent in socioeconomically challenged populations around the world, and because large sample sizes are needed to test nutrigenomic relationships, Kaput and his colleagues are pushing for an international effort to study micronutrient needs based on differing genetic makeups among different ancestral groups.

Bruce Ames, a molecular biologist at Children’s Hospital Oakland Research Institute in California, has documented a number of polymorphisms in genes that affect the binding of coenzymes, some of which are essential vitamins. “With these types of evidenced-based findings within the nutrigenomic framework, I believe we’ll have more ammunition to convince government and public health officials to tackle the issue of vitamin deficiency around the world,” Kaput says. “With this more targeted approach, we’re more likely to see political and economic forces fall in place to solve the problem. . . . Although the complexities are substantial, I believe nutrigenomic approaches offer the best hope for understanding the molecular processes that maintain health and prevent disease.”

For Fenech, one of the key objectives of nutrigenomics for society is to diagnose and nutritionally prevent DNA damage on an individual-by-individual basis. He has devised the concept of the Genome Health Clinic, a new mode of health care based on the diagnosis and nutritional prevention of DNA damage and the diseases that result therefrom. In recent years, a number of nutritional/metabolic/diagnostic testing companies such as Genova and MetaMetrix have started to sell genomic profiling tests to help guide decision making around dietary supplements. With the increasingly lower pricings for analyzing SNPs in individuals, the population-level potential for dietary optimization based on nutrigenomic approaches seems truly awesome. Even in the absence of information on an individual’s genotype, it is practical to use nutrition-sensitive genome damage biomarkers, such as the micronucleus assay, to determine whether dietary and/or supplement choices are causing benefit or harm to a person’s genome.

Says Fenech, “In the near future, instead of diagnosing and treating diseases caused by genome or epigenome damage, health care practitioners may be trained to diagnose and nutritionally prevent or even reverse genomic damage and aberrant gene expression. Nutrigenomics will help usher in the development of new functional foods and supplements for genome health that can be mixed and matched so that overall nutritional intake is appropriately tailored to an individual’s genotype and genome status.”

Research presented at a November 2007 meeting suggests that inositol (a member of the B vitamin family found in grains, seeds, nuts, brewer’s yeast, and many other foods) and its derivative inositol hexaphosphate (IP6) help protect against genetic damage from UVB and other radiation. In one experiment, human skin cells treated with IP6 were less likely than untreated cells to undergo apoptosis, indicating that they had less irreparable DNA damage. In another experiment, mice genetically engineered for a propensity to skin cancer drank water containing 2% IP6. Tumors developed in 23% of these mice compared with 51% of mice that did not receive IP6. Use of a cream containing inositol and IP6 also protected against tumor development in mice exposed to UVB radiation. The researchers suggest that people who are regularly exposed to ionizing radiation, such as airline pilots, frequent fliers, or people who handle radioactive materials, might take IP6 prophylactically to prevent possible long-term effects of exposure.

Source: Shamsuddin AM. Paper presented at: American Association for Cancer Research Centennial Conference on Translational Cancer Medicine: From Technology to Treatment; Singapore; 4–8 November 2007.

An article published in the October 2007 issue of the *British Journal of Nutrition* warns that fortifying flour with folic acid—a move intended to prevent neural tube defects among mothers who eat the flour—may lead to numerous unforeseen health problems. Unlike the natural folates found in leafy green vegetables, which are digested in the gut, synthetic supplements are now believed to be metabolized in the liver. The study authors hypothesize that the liver becomes saturated, and unmetabolized folic acid enters the blood stream, where it can contribute to leukemia, arthritis, colorectal cancer, and ectopic and multiple pregnancies. Other recent findings on a potential link between supplementation and colorectal cancer are examined in two commentaries in the November 2007 issue of *Nutrition Reviews*. The new data follow on the heels of the U.K. Food Standard Agency’s May 2007 approval of the addition of folic acid to flour. The United States, Canada, and Chile also currently fortify flour with folic acid, and the policy is being considered for implementation in Australia, New Zealand, and Ireland.

Sources: Wright AJA, et al. 2007. Folic acid metabolism in human subjects revisited: potential implications for proposed mandatory folic acid fortification in the UK. Br J Nutr 98(4):667–675; Kim Y-I. 2007. Folic acid fortification and supplementation—good for some but not so good for others. Nutr Rev 65:504–511; Solomons NW. 2007. Food fortification with folic acid: has the other shoe dropped? Nutr Rev 65:512–515.

Antioxidants are known for their ability to slow the oxidation that damages cells. But the human body doesn’t derive the same level of benefit from all antioxidants. Recently nutritionists with the USDA Agricultural Research Service measured the plasma antioxidant capacity (AOC) of study subjects following a single meal of blueberries, cherries, dried plums, dried plum juice, grapes, kiwis, or strawberries. They reported in the April 2007 *Journal of the American College of Nutrition* that blueberries, grapes, and kiwifruit yielded the greatest increases in plasma AOC. Plums—despite their high antioxidant content—did not raise plasma AOC levels, probably because chlorogenic acid, the antioxidant in which they are richest, is not readily absorbed by humans.

Norwegian researchers showed in the August 2007 issue of the *Journal of Nutrition* that anthocyanins from bilberries and black currants reduced levels of transcription factor NF-κB in cultured cells. NF-κB orchestrates a wide range of inflammatory responses. In humans, anthocyanin supplementation decreased interleukin-8, IFN, and normal T cell expression by 25%, 25%, and 15%, respectively, over placebo. The authors suggest that anthocyanins and/or their metabolites may serve as redox buffers capable of suppressing oxidative stress and thereby dampen the inflammatory response by direct reactive oxygen species scavenging.

Sources: Prior RL, et al. 2007. Plasma antioxidant capacity changes following a meal as a measure of the ability of a food to alter in vivo antioxidant status. J Am Coll Nutr 26(2):170–181; Karlsen A, et al. 2007. Anthocyanins inhibit nuclear factor-B activation in monocytes and reduce plasma concentrations of pro-inflammatory mediators in healthy adults. J Nutr 137:1951–1954.

## Figures and Tables

**Figure:**
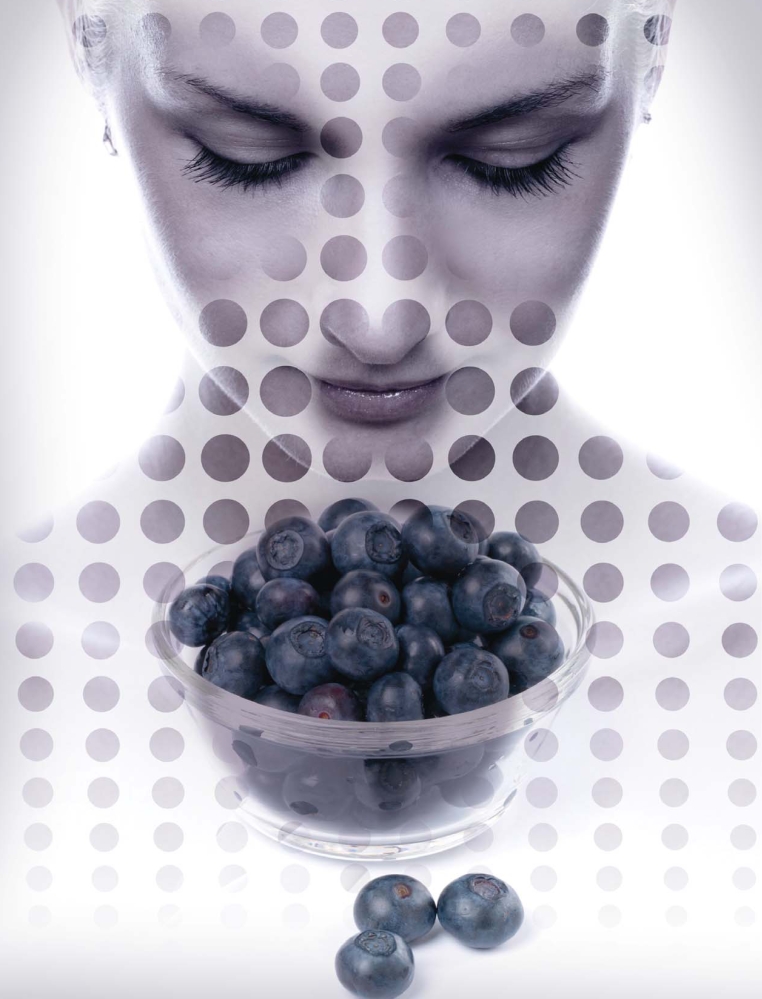


**Figure:**
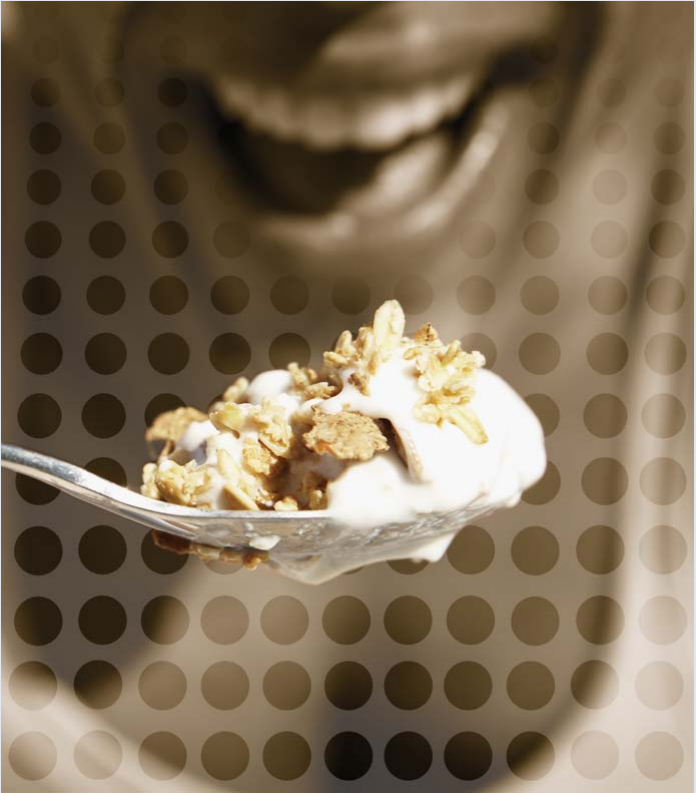


**Figure:**
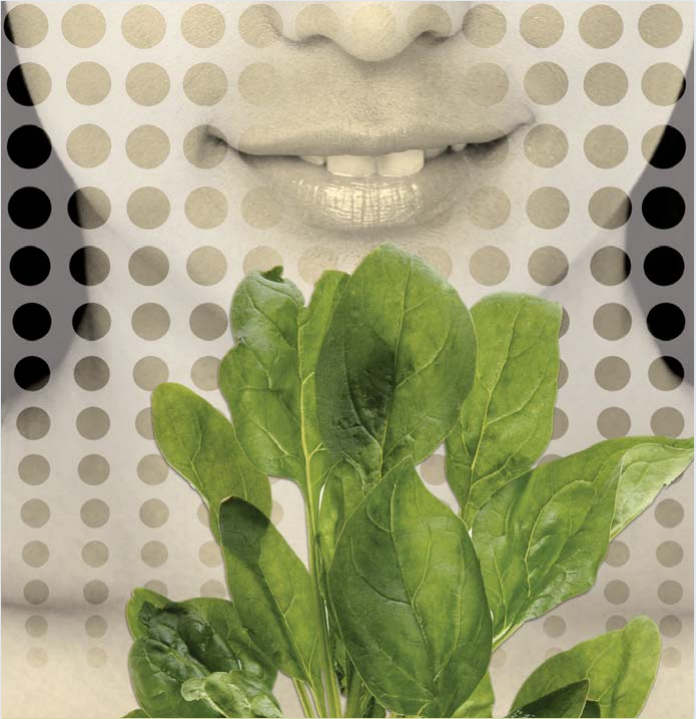


**Figure:**